# Emma Wilson Mooers (1858–1911): die Neuropathologin an Aloys Alzheimers Seite

**DOI:** 10.1007/s00115-020-00979-w

**Published:** 2020-08-11

**Authors:** Hans Förstl

**Affiliations:** Klinik und Poliklinik für Psychiatrie und Psychotherapie, Technische Universität München, Ismaninger Str. 22, 81675 München, Deutschland

## Gruppenfoto mit Damen

**Figure 1:**
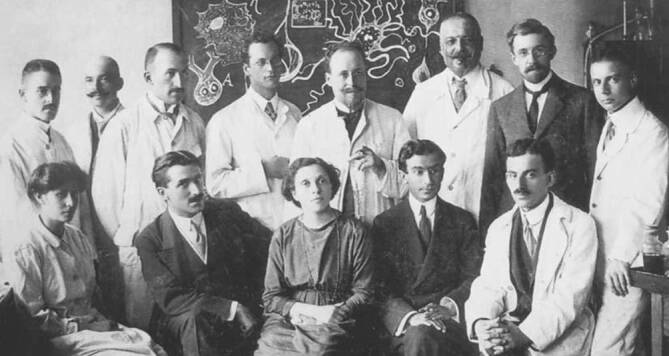

Das bekannteste Bild aus seinem Münchner Labor zeigt Aloys Alzheimer selbst und die junge, internationale Elite der Hirnforschung, nämlich Nicolas Achúcarro, Rudolf Allers, Francesco Bonfiglio, Ugo Cerletti, Friedrich Heinrich Lewy, Fritz Lotmar, Gaetano Perusini, Stefan Rosenthal und links vorne Adele Grombach (Abb. 1). Wer aber ist die selbstbewusst in der Mitte sitzende und von den Herren umringte Dame im dunklen Kleid, die auf der Fotografie üblicherweise mit einem Fragenzeichen oder als „unbekannt“ bezeichnet wird? Sie war die Koleiterin des Anatomischen Labors, Emma Wilson Mooers, geboren 1858 in Greendale, Wisconsin.

## Biografie einer Forscherin

Im Jahr 1884 schloss Emma W. Mooers das Medizinstudium an der University of Michigan in Ann Arbor ab. Sie war eine von mehreren Ärztinnen deren Namen zwei Jahre später in einem launigen Bericht der *New York Tribune* und des *Ann Arbor Couriers* Erwähnung finden: „Mehr als 50 Alumni der University of Michigan nahmen letzte Nacht am Dinner der New York Association … teil. Ein neuer, aber nichtsdestoweniger angenehmer Zug war die Teilnahme von Absolventinnen, zehn Damen … aus Ann Arbor, welche mit der Anmut femininer Vollendung und Errungenschaften zum Glanz des Ereignisses beitrugen“ [1]. Dies schien offensichtlich noch der Erwähnung wert, obwohl Frauen das Medizinstudium an einigen US-amerikanischen Lehreinrichtungen bereits seit Mitte des 19. Jahrhunderts möglich war und damit 50 Jahre früher als an Universitäten in Deutschland [2]. Danach ist Dr. Emma D. Mooers als Mitglied in den Berichten der American Public Health Association aufgeführt. 1888 wohnt sie in Arlington, Massachusetts. 1898 wird sie Assistenzärztin am Northampton Insane Hospital. 1899 wird sie in die American Medico-Psychological Association aufgenommen.

Im Jahr 1902 taucht der Name Emma Wilson Mooers im Zusammenhang mit einem Skandal im *Journal of the American Medical Association* auf [11]: Eine Betrügerin hatte sich mit der Behauptung, sie sei Dr. Emma W. Mooers und ihre Unterlagen wären verbrannt, beim Sekretär der University of Michigan falsche Dokumente erschlichen. Die falsche Dr. Mooers praktizierte zunächst im nördlichen Michigan, danach in Chicago und schließlich in Colorado, wo sie gefasst werden konnte. Ein Kollege hatte sich bei der University of Michigan über sie beschwert. Dort aber war bekannt, dass die richtige Dr. Mooers zu der Zeit als Pathologin am McLean Hospital, der psychiatrischen Klinik von Harvard, in Waverley, Mass., arbeitete. „Der ganze Vorgang belastete die richtige Dr. Mooers erheblich, deren Arbeit stets von höchster Qualität und deren Verhalten äußerst professionell war“ [11].

Im Jahr 1903 veröffentlichte Emma W. Mooers eine ausführliche Studie im *Boston Medical and Surgical Journal* über eine vermutlich bakterielle Meningoenzephalomyelitis mit makropathologischen und histologischen Abbildungen, in der sie auch mehrere deutschsprachige Arbeiten zitierte [6]. 1904 erschien ihre Arbeit über den amnestischen Symptomenkomplex bei Neurosyphilis. Sie war die erste *Forscherin* am McLean Hospital [7].

## München und Harvard

Der Jahresbericht Michigan Alumnus vermerkt 1904 Emma Mooers sei nach Übersee gegangen und nun über die Adresse c/o Brown, Shipley & Co, 123 Pall Mall, London, zu erreichen. Im Wintersemester 1905/1906 ist sie im Gasthörerverzeichnis der Münchner Universität verzeichnet; sie beschäftige sich mit Psychiatrie und Anatomie.

Im ausführlichen Jahresbericht 1906/1907 der Königlich Psychiatrischen Klinik in München schreibt Kraepelin auf der ersten Seite „in die Reihe der wissenschaftlichen Assistenten trat zunächst Dr. Plaut, dann die Herren Rüdin und Isserlin, endlich Frau Dr. Mooers … Frau Dr. Mooers unterstützte Dr. Alzheimer in der Leitung des anatomischen Laboratoriums“ [4]. Kraepelin akzeptierte keine Mitarbeiter, die nicht ausreichend Deutsch sprachen. Zwei bedeutende kanadische Psychiater besuchten die Münchner Klinik im Sommer 1907 und erstatteten ausführlich Bericht im *American Journal of Insanity*. C.K Clarke, Professor für Psychiatrie an der Universität Toronto, erwähnt „Drs. Gudden, Moers (!), Plaut, Weiler und andere haben bereits wohlverdienten Ruhm erworben und die Arbeiten dieser enthusiastischen Bande haben die psychiatrische Wissenschaft auf bemerkenswerte Weise bereichert und Licht auf die verzwicktesten Probleme geworfen, mit denen wir uns beschäftigen müssen“ [3]. Dr. Ryan aus Kingston, Ontario, schrieb: „Dr. Mooers, eine ÄRZTIN (‚a lady physician‘) aus Amerika, ist eine bekannte Dozentin und Meisterin der Technik“ [8]. Auf der ersten Seite des Jahresberichts 1908/1909 erwähnt Kraepelin, von den wissenschaftlichen Assistenten sei Frau Mooers ausgeschieden, um nach Amerika zurückzugehen [5]. Ein Vergleich der Zeiten, zu denen die fotografierten Forscher in München arbeiteten und die Sitzordnung der Kollegen um Emma W. Mooers herum, legen den Schluss nahe, es könne sich um Mooers’ Abschiedsfoto aus Alzheimers Labor handeln (Abb. 1).

**Figure 2:**
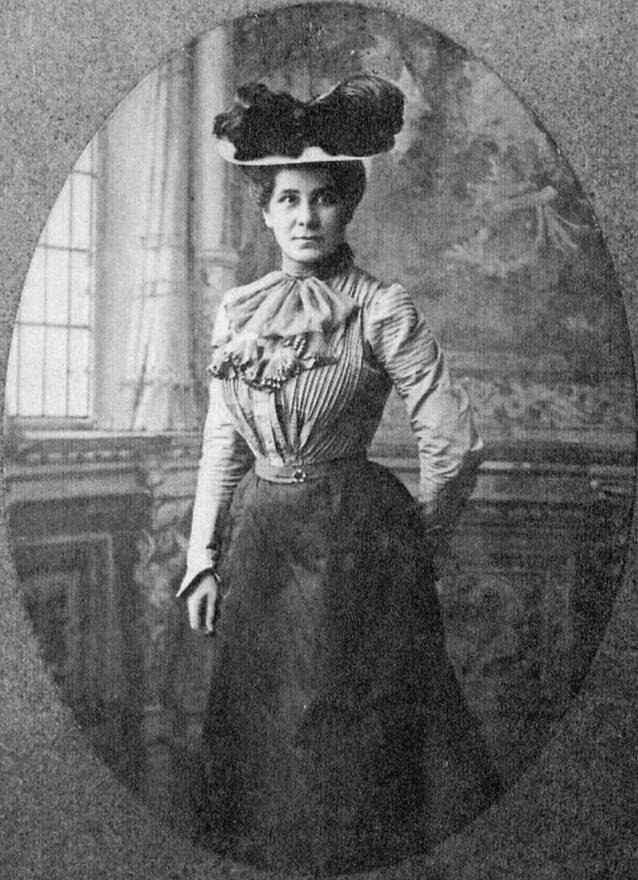

Die Abb. 2 zeigt Emma W. Mooers in den USA, würdevoll und noch besser gekleidet als kurz davor in München. Am 20.07.1910 wurde sie zur „Kuratorin“ („Custodian“) der Neuropathologischen Sammlung ernannt und war damit die zweite Frau an der Harvard Medical School, wenngleich ohne richtige Fakultätszugehörigkeit [9]. Wiederholte Hinweise, dass sie einen Doktortitel besitze, fanden keine Resonanz auf Seiten der Universitätsleitung. Ihr hochgeachteter Kollege Elmer Ernest Southard, Bullard Professor für Neuropathologie, schrieb an den Universitätspräsidenten, die Bezeichnung „Kuratorin“ bringe die wahre Bedeutung der Position und Mooers’ Rolle in der Forschung nicht gebührend zum Ausdruck. Mooers selbst fand die Bezeichnung unwürdig. Am 29.09.1910 schrieb Universitätspräsident Lowell, er sei gerne bereit die Bezeichnung dergestalt zu verändern, dass Mooers zufrieden damit sei, „solange dies nicht beinhalte, dass sie – oder andere Frauen – berechtigt seien Fakultätsmitglieder zu werden“ [9].

Am Samstag dem 13.05.1911 verletzten sich Mooers und Southard bei der Autopsie eines Mannes mit einer hochvirulenten Streptokokkentonsillitis, die damals viele Todesopfer forderte. Emma Mooers starb am 31.05.1911 mit 52 Jahren an einer Streptokokkensepsis und Meningitis. Nachrichten von ihrem Tod erschienen in *Science*, dem *British Medical Journal* und diversen Tageszeitungen, eine „Märtyrerin der Wissenschaft“. Southard erholt sich nach schwerer Krankheit und erlag 1920 einer Pneumonie. Er zitierte Mooers’ Beitrag zu den serologischen Arbeiten Plauts [10]. Andere erwähnen ihre Arbeiten zur Neurosyphilis und einer gemeinsam mit Minkovski entwickelten Färbemethode.

Auf Mooers’ Grabstein steht „Assistant in Psychiatric Clinic Munich 1905–1911, Custodian of the Harvard Neuropathological Collection 1910–1911; a devoted and discerning worker in the technic and science of neuropathology“ (Countway Library). Die Verbindung zur Münchner Klinik hatte sie also beibehalten. Sie war zu früh gekommen und zu früh gegangen, zu früh um erfolgreich antibiotisch behandelt zu werden, zu früh um als Frau die große Karriere zu machen und in Erinnerung zu bleiben. Selbst die Erinnerung an sie als zentrale Figur eines Gruppenportraits, das vermutlich ihr zu Ehren gemacht wurde, ging verloren.
